# Nickel and Cobalt Release From Hairdressing Tools in German Barbershops

**DOI:** 10.1111/cod.70031

**Published:** 2025-09-07

**Authors:** Cara Bieck, Kirsten Koopmann, Antje Alberts, Valeska Buder, Grita Schedlbauer, Albert Nienhaus, Christoph Skudlik, Swen Malte John

**Affiliations:** ^1^ Department of Dermatology, Environmental Medicine and Health Theory Osnabrück University Osnabrück Germany; ^2^ Department of Occupational Medicine, Hazardous Substances and Health Sciences Statutory Accident Insurance for the Health and Welfare Services Hamburg Germany; ^3^ Institute for Interdisciplinary Dermatological Prevention and Rehabilitation (iDerm) Osnabrück University Osnabrück Germany; ^4^ Institute for Health Services Research in Dermatology and Nursing (CVcare) University Medical Center Hamburg‐Eppendorf (UKE) Hamburg Germany

**Keywords:** allergy, barber, cobalt, Germany, hairdressing, nickel, spot test, tool

## Abstract

**Background:**

Nickel and cobalt release from tools has recently been evidenced in German hairdressing salons. Comparable data were not available for German barbershops.

**Objectives:**

Screening of tools for nickel and cobalt release.

**Methods/Materials:**

One hundred and forty‐one tools were tested in six barbershops located in Lower Saxony, Germany. A nickel spot test (reagent: dimethylglyoxime [CAS no. 95‐45‐4]) and a cobalt spot test (reagent: nitroso‐r salt [disodium‐1‐nitroso‐2‐naphthol‐3,6‐disulphonate; CAS no. 525‐05‐03]) were used.

**Results:**

35 of 141 tools overall (24.8%) released nickel and 3 of 141 tools overall (2.1%) released cobalt. Nickel release was found in 10 of 57 hair clippers, 9 of 13 tweezers, 8 of 11 sectioning clips, 3 of 14 straight shavers, 2 of 32 scissors, 2 of 4 tail combs and 1 of 2 shaving brushes. Cobalt release was detected in 2 of 14 straight shavers and 1 of 11 sectioning clips.

**Conclusions:**

Tools in German barbershops have been identified as occupational sources of nickel and cobalt exposure. Nickel release was observed more frequently. Therefore, it is recommended that compliance with the EU nickel regulation is observed more strictly. In addition, the importance of measures to protect the skin at work should be communicated to barbers, e.g., within health pedagogical measures.

## Graphical abstract

Screening for nickel and cobalt release from tools in German barbershops. 35 of 141 tools (24.8%) released nickel. 3 of 141 tools (2.1%) released cobalt.

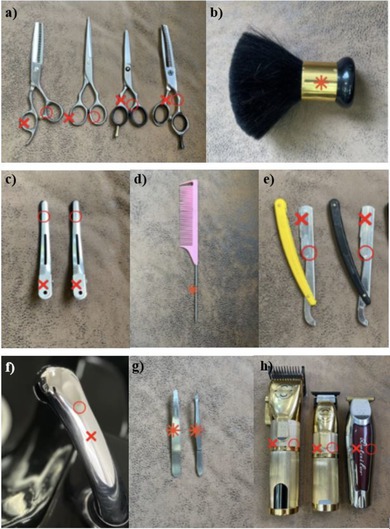

## 1 Introduction

Nickel and cobalt have consistently ranked amongst the most prevalent contact allergens in recent years, which accounts for Germany [1] as well as on a European level [2]. The European Union (EU) regulates the use of nickel in metallic goods through the Registration, Evaluation, Authorisation and Restriction of Chemicals (REACH) Regulation (EC 1907/2006) [3]. As of right now, there has been no comparative legislative restriction regarding the use of cobalt in metallic goods. In the German hairdressing trade, more precisely in conventional hairdressing salons (i.e., hairdressing salons conducting all hairdressing services according to the German educational regulations, including hairdressing services in male and female customers), nickel and cobalt release was found in two current studies; sectioning clips, tail combs, tweezers, hair clips, crochet hooks, and straight razors have been identified as nickel and/or cobalt releasing [4, 5]. Data on nickel and/or cobalt release from metallic work tools in German barbershops—which belong to the hairdressing trade and can be classified as hairdressing salons mostly specialising in hairdressing services for men (including classic barber services, such as shaving beards)—are presently lacking. One recent study, which was conducted in barbershops in the United States of America (USA) where no legislative regulation regarding the use of nickel in metallic goods is in place, however, provides data on nickel release from tools used in barbershops. Peterson and Hylwa [6] found nickel release in 10 of 192 metal tools (5.2%)—these include scissors, trimmers, hairdressing chairs, cape clips, and nail clippers.

Workers in barbershops (which are from now on called *barbers* in this paper) are potentially exposed to occupational skin strain which might be comparable to the skin strain of hairdressers working in conventional hairdressing salons [7]. This high level of occupational skin strain in barbers is a result of frequently working in wet conditions and coming into touch with detergents and salon chemicals, which results in a weakened skin barrier homeostasis concomitant with the induction of a proinflammatory skin milieu that facilitates penetration of, and sensitisation to, harmful substances [8, 9]. The risk of developing occupational allergic contact dermatitis of the hands is thus elevated [10]. Considering the individual suffering as well as the socio‐economic consequences—including high illness costs—that occupational hand eczema entails [11], the development of occupational hand eczema should be prevented.

Given this context, the current study seeks to screen hairdressing tools used in German barbershops for nickel and cobalt release in order to identify potentially relevant sources of exposure. Perspectively, the findings shall benefit prevention and treatment of contact dermatitis, especially occupational allergic contact dermatitis caused by nickel and/or cobalt, in the hairdressing trade.

## 2 Methods/Materials

Nickel and cobalt spot testing was conducted in six randomly chosen barbershops (i.e., hairdressing salons specialising on hairdressing services for men with the self‐denomination ‘barbershop’) located in Lower Saxony, Germany from February to March 2024. In the opportunity sample, all available metal tools and tools with metallic parts with which barbers may potentially have relevant skin contact were included, i.e., hairdressing tools with intended prolonged skin contact according to the definition by the European Chemicals Agency (ECHA): skin contact with nickel of more than (i) 10 min on three or more occasions within 2 weeks or (ii) 30 min on one or more occasions within 2 weeks [12]. Self‐reported data about the age, price and manufacturer of the tested tools were obtained by asking the barbers.

**FIGURE 1:**
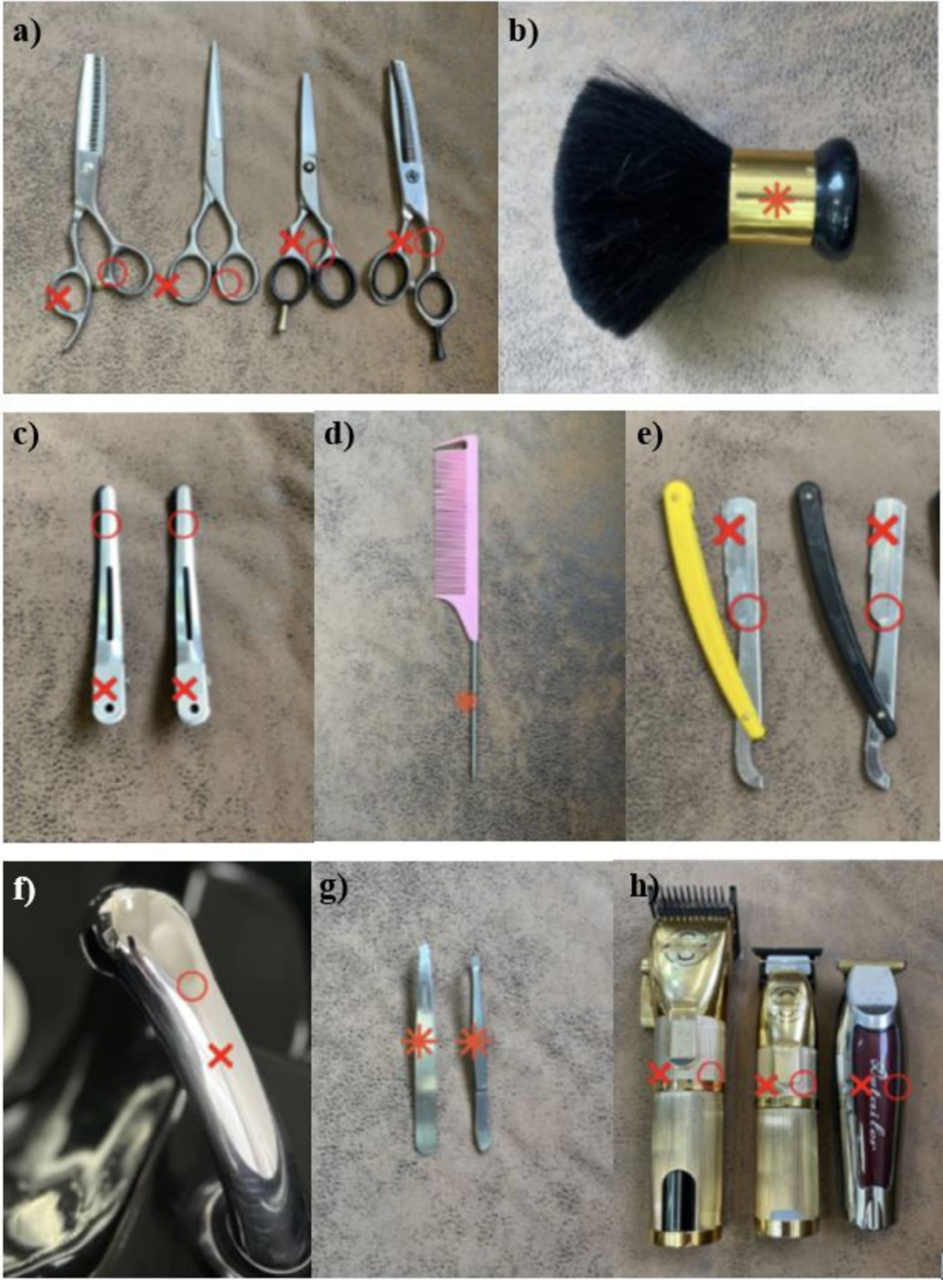
Tested tools alongside with pre‐determined testing sites. Nickel spot test testing sites are marked with an x, whereas cobalt spot test testing sites are marked with a circle (o). Asterisks (*) indicate testing sites where nickel and cobalt spot tests were performed on the corresponding opposite sides of the metal tool. (a) scissors for manually cutting hair, (b) shaving brush used for the application of shaving cream for wet shaving, (c) sectioning clips for sectioning hair, (d) tail comb for parting and sectioning hair, as well as for precision styling, (e) straight razor for shaving after applying shaving cream, (f) hand shower used for wetting hair and rinsing hairdressing products such as shampoo and hair colour, (g) tweezers (customary models) for plucking hair and (h) hair clippers for trimming or shaving hair to a short length.

A commercially available dimethylglyoxime (DMG; CAS no. 95‐45‐4) test solution (nickel spot test) and a nitroso‐r salt (disodium‐1‐nitroso‐2‐naphthol‐3,6‐disulphonate; CAS no. 525‐05‐03) test solution (cobalt spot test) was used. These are semi‐quantitative tests that are evaluated qualitatively in this study. To guarantee equal testing, the testing sites were predetermined (Figure 1). The testing spots were typically 0.5 cm^2^ in size, depending on the tool. According to the dimension of the tools and the locations where they come into contact with the skin, a 2.0 × 1.25 cm strip of common medical adhesive tape was applied between the relevant testing sites to ensure that the test results were not contaminated by the overlap of the two different test solutions. Whenever possible, a greater spacing than 1.25 cm was maintained between different testing regions. If possible, testing with the two solutions was carried out on the other side of the tool.

The examinations followed the testing solution producers' guidelines. Two drops (approximately 50 μL) of the test solution were placed to a new conventional cotton swab from pure cotton wool. The cotton swab was then rubbed against the selected testing spot for a maximum of 60 s. If a relevant colour change was detected before this time elapsed, the experiment was ended. A positive nickel test reaction was indicated by a colour shift from the previously clear nickel test solution to a reddish‐pink colour, whilst a negative test reaction was indicated by no colour change in the test solution. A positive cobalt test reaction was detected by a colour shift from yellow to reddish‐brown in the test solution, whilst a negative reaction was shown by no colour change of the yellow test solution. Inconclusive responses to the tests were discovered by colour shifts different than the target colour for both test solutions (e.g., black, green, blue) and recorded separately, without being construed as positive or negative, as has been recommended by Wennervaldt et al. [13]. When analysing the colour shift, the cotton swabs were placed in front of a white surface to improve colour recognition. The tools were tested in their original state, without any cleaning or manipulation prior to the testing. The testing protocol described above has been used before in comparable studies and was proven practical [4, 5, 14].

## 3 Results

In total, 141 beauty tools were tested for nickel and cobalt release. The price and the age of the 141 tested tools ranged from 1 to 600 € (median: 84.5 €; mean: 100.7 €) and 1 to 10 years (median: 1; mean: 1.6). For 24 of 141 tools (17.0%), no manufacturer could be noted; the remaining 117 tools were produced by 23 different manufacturers. The price of 19 tools was not documented due to missing information.

The nickel spot test showed that 35 of 141 beauty tools overall (24.8%) released nickel and the cobalt spot test showed that 3 of 141 beauty tools overall (2.1%) released cobalt (Table 1). Nickel release was found in 10 of 57 hair clippers (17.5%), 9 of 13 tweezers (69.2%), 8 of 11 sectioning clips (72.7%), 3 of 14 straight razors (21.4%), 2 of 32 scissors (6.3%), 2 of 4 tail combs (50.0%) and 1 of 2 shaving brushes (50.0%). Nickel‐releasing hairdressing tools were found in every visited barbershop. Cobalt release was detected in 2 of 14 straight razors (14.3%) and 1 of 11 sectioning clips (9.1%). Of 3 cobalt‐releasing beauty tools, 2 simultaneously released nickel (66.7%); 1 sectioning clip and 1 straight razor. Characteristics of the tools with positive test results (i.e., age, price and manufacturers) are displayed in Table 1.

**TABLE 1:** Positive nickel and cobalt test results of the tested hairdressing tools in the present study alongside with characteristics of the tools with positive test results (age, price and manufacturers).

Metal tools	Nickel positive					Cobalt positive				
	%	*n*/*n*_total_	Age range (M ± SD) in years	Price range (M ± SD) in Euro	No. of manufacturers	%	n/*n*_total_	Age range (M ± SD) in years	Price range (M ± SD) in Euro	No. of manufacturers
Scissors	6.3	2/32	1.0 (1.0 ± 0)	10.0–40.0 (25.0 ± 21.2)	1 + x	0	0/32	n/a	n/a	n/a
Hand showers	0	0/8	n/a	n/a	n/a	0	0/8	n/a	n/a	n/a
Tail combs	50.0	2/4	1.0 (1.0 ± 0)	5.0 (5.0 ± 0)	1	0	0/4	n/a	n/a	n/a
Straight razors	21.4	3/14	1.0 (1.0 ± 0)	2.0–15.0 (10.7 ± 7.5)	2	14.3	2/14		2.0–15.0 (8.5 ± 9.2)	2
Shaving brush	50.0	1/2	2.0 (2.0 ± 0)	x	1	0	0/2	n/a	n/a	n/a
Sectioning clips	72.7	8/11	1.0–2.0 (1.75 ± 0.5)	1.0 + x (1.0 ± 0)	x	9.1	1/11	1.0 (1.0 ± 0)	1 (1.0 ± 0)	x
Tweezers	69.2	9/13	1.0 (1.0 ± 0)	2.0–3.0 (2.4 ± 0.5)	1 + x	0	0/13	n/a	n/a	n/a
Hair clippers	17.5	10/57	1.0–2.0 (1.1 ± 0.3)	100.0–300.0 + x (167.8 ± 64.2)	3	0	0/57	n/a	n/a	n/a
All	24.8	35/141	1.0–2.0 (1.2 ± 0.4)	1.0–300.0 + x (60.2 ± 85.6)	8 + x	2.1	3/141	1.0 (1.0 ± 0)	1.0–15.0 (6.0 ± 7.8)	2 + x

*Note*: +x indicates that a share of tools in this line could not be attributed to a manufacturer or price and thus a more exact specification is not possible.

Abbreviations: M, Mean; n/a, not applicable; SD, standard deviation.

Inconclusive test results (i.e., a colour change of the spot test different to the target colour of the corresponding test solution) were seen in 1 of 11 sectioning clips (9.1%) with the cobalt spot test, characterised by a colour change of the test solution to green. The eight tested hand showers did not release nickel or cobalt. Retesting of 17 (12.1%) randomly selected sample of the previously tested tools with a time lag of 3 weeks revealed a reproducibility of the results of 100%.

## 4 Discussion

The presented study is—to the best of our knowledge—the first to screen hairdressing tools in barbershops in Germany for nickel and cobalt release. The results demonstrate that several tools commonly used in both hairdressing salons and barbershops might pose a risk of mainly nickel but also cobalt exposure. As also found in previous studies in hairdressing salons [4, 5, 15], sectioning clips, tweezers and straight razors appear most frequently amongst nickel releasing tools (Table 2). Straight razors and sectioning clips, which have been found as cobalt releasing tools in German hairdressing salons [5], also released cobalt in the German barbershops visited during the presented examination. One striking category of tools are scissors: Whereas scissors were not identified as nickel releasing in German hairdressing salons [4, 5], they were identified as nickel releasing in the present study as well as in current examinations in American hairdressing salons and barbershops [6, 15]. This emphasises that also tools in the more expensive categories (e.g., scissors) cannot reliably be seen as safe in terms of nickel release. Thyssen et al. [16] came to a similar conclusion in 2009, when out of 200 scissors tested in the Danish hairdressing trade, the only one that released nickel was in the expensive price range. Therefore, it is important to test materials regardless of price (or brand reputation), especially if a hairdressing tool is suspected to be the cause of occupational allergic contact dermatitis.

**TABLE 2:** Results of previously conducted studies regarding nickel and cobalt release from tools in hairdressing salons and barbershops in Denmark, Germany and the United States of America (USA) in comparison to the presented study.

Tools	Thyssen et al. [16]		Symanzik et al. [4]		Symanzik et al. [5]				Peterson and Hylwa [6]		Chan and Hamann [15]		Presented study			
	Hairdressing salons in Denmark		Hairdressing salons in Germany		Hairdressing salons in Germany				Barbershops in the USA		Hairdressing salons in the USA		Barbershops in Germany			
	Nickel positive		Nickel positive		Nickel positive		Cobalt positive		Nickel positive		Nickel positive		Nickel positive		Cobalt positive	
	%	*n*/*n*_total_	%	*n*/*n*_total_	%	*n*/*n*_total_	%	*n*/*n*_total_	%	*n*/*n*_total_	%	*n*/*n*_total_	%	*n*/*n*_total_	%	*n*/*n*_total_
Scissors	0.5%	1/200	0	0/62	0	0/116	0	0/116	1.8	1/57	3.6	1/28	6.3	2/32	0	0/32
Hand showers	n/t	n/t	0	0/13	0	0/20	0	0/20	n/t	n/t	n/t	n/t	0	0/8	0	0/8
Tail combs	n/t	n/t	7.4	2/27	8.9	4/45	0	0/45	n/t	n/t	0	0/3	50.0	2/4	0	0/4
Straight razors	n/t	n/t	n/t	n/t	15.6	5/32	3.1	1/32	0	0/19	0	0/2	21.4	3/14	14.3	2/14
Sectioning clips	n/t	n/t	17.8	8/45	44.0	33/75	2.7	2/75	n/t	n/t	35.7	5/14	72.7	8/11	9.1	1/11
Tweezers	n/t	n/t	64.7	11/17	60.0	24/40	7.5	3/40	0	0/12	n/t	n/t	69.2	9/13	0	0/13
Hair clippers	n/t	n/t	n/t	n/t	n/t	n/t	n/t	n/t	0	0/30	50.0	5/10	17.5	10/57	0	0/57
Shaving brush	n/t	n/t	n/t	n/t	n/t	n/t	n/t	n/t	n/t	n/t	n/t	n/t	50.0	1/2	0	0/2
Others	53.8	7/13	n/t	n/t	44.2	65/147	2.7	4/147	12.2	9/74	100	3/3	n/t	n/t	n/t	n/t
All	3.8	8/213	9.2	21/229	27.6	131/475	2.1	10/475	5.2	10/192	27.0	24/89	24.8	35/141	2.1	3/141

Abbreviations: n/t, not tested; USA, United States of America.

The overall share of nickel and cobalt releasing tools in German barbershops seems to be corresponding to the share of nickel and cobalt releasing tools in German and American hairdressing salons [4, 5, 15], whereas a lower share of nickel releasing tools has been reported in American barbershops [6]. This finding needs to be evaluated with caution, as the range of tested tools within the different studies is not fully comparable. This might be especially relevant for the comparison of nickel and cobalt releasing tools in German hairdressing salons and German barbershops. Even though it is not possible to definitely explain the reasons for the differences regarding the nickel and cobalt releasing tools, it might be surmised that these differences result from different materials being used for manufacturing the respective tools. It, however, is noteworthy that a higher proportion of nickel releasing tools was found in a country with a nickel regulation (i.e., Germany) compared to tools in a country without a nickel regulation (i.e., the USA). This, again, could potentially be led back to differing metal tool samples in the different studies but also to differing materials used for the respective tools. Nevertheless, the results of the present study allow the conclusion that workers in German barbershops are subjected to nickel and cobalt releasing tools at an extent which is comparable to German hairdressing salons. Further, the findings stress the need for consequent monitoring of compliance with the nickel regulation in terms of nickel contents in tools with intended prolonged skin contact according to the ECHA definition by the respective authorities.

Other studies in which positive nickel test results were obtained also included tools or metal applications with which employees do not have intended prolonged skin contact according to the definition mentioned above, such as cape clips or hairdressing/barbershop chairs [6, 15]. Although these are not of high interest in terms of exposure for the barber or hairdresser, they might be more important in terms of protecting the clients (who might have prolonged skin contact with the respective materials). Such objects could be subject to a closer monitoring in future studies. The metal tools tested in this study were tested in barbershops; yet, they are likely also utilised in hairdressing salons. Consequently, the results of the present examination can be deemed relevant for the entire hairdressing trade. Further, there might be an overlap in tools used in hairdressing salons/barbershops and in cosmetic salons. In a recent study by Symanzik et al. [14], nickel and cobalt release was detected in tweezers and other tools in cosmetic salons. Especially tweezers pose an example for a tool used in cosmetic salons, barbershops and hairdressing salons and showcase the need to protect workers in the beauty sector as a whole.

It is also important to note that barbers are predominantly male and that the development of a nickel allergy in the private area is less of a problem in men, as it is often caused by factors as ear piercing during childhood or the wearing of fashion jewellery containing nickel and cobalt (mainly reported in women) [17]. Nevertheless, existent sensitisations are not irrelevant in an occupational context, as afresh skin contact with the allergen can lead to elicitation and consequently recurring allergic reactions and occupational allergic contact dermatitis [17]. As described in previous investigations, the relation between nickel and cobalt sensitisation should be noted (i.e., nickel being an adjuvant in cobalt sensitisation) [5]. Due to this, especially co‐exposure to nickel and cobalt should be avoided. This is especially important in the light of the skin strain in barbers due to the facilitated penetration of allergens in pre‐damaged skin [8, 9]. Thus, it seems to be imperative to provide health education, especially in terms of primary prevention, also to barbers in order to keep the skin in the best possible condition and ideally prevent sensitisation. If barbers are sensitised, they should receive health education in terms of secondary prevention as soon as possible to initiate adequate allergen avoidance, which is to date the only effective measure for dealing with allergic contact dermatitis; an effective causal therapy for type IV hypersensitivity (contact allergy) does not yet exist. In view of the consequences of hand eczema, in the worst case caused by an unavoidable allergen at work, including sick leave and, in the worst case, change of profession or job loss [11], this is not only very relevant for the employee, but also in the best interest of the employer. Clearly, by a careful choice of tools, nickel exposure in hairdressing would be completely avoidable. In this, it should be borne in mind that hand eczema often sets on during apprenticeship or during early career stages [10]. Therefore, health educative measures about the risk potential of metal tools must not be neglected and education about preventive measures should start as early as possible. Given the large (and continuing) increase in barbershops—not only in Germany but also in other countries—over the last few years, the results of the present work might become even more relevant in the future.

A limitation of this study is that it comprises only a small opportunity sample of hairdressing tools in a limited number of German barbershops during a limited window of time. Therefore, the results should be interpreted rather as orientating and not be generalised for the situation in all German barbershops. The methodology presented in this paper could be applied to a larger sample size in future studies, which would ascertain the generalisability of the results. It also should critically be mentioned that the nickel and cobalt spot tests are only suitable to a limited extend. Nickel spot testing by dimethylglyoxime and cobalt spot testing by nitroso‐R‐salt represent semi‐quantitative methods, which can detect relevant emission of nickel and/or cobalt ions with regard to sensitisation and elicitation, as described in previous studies [18, 19, 20, 21]; though, the exact amount of nickel and/or cobalt release remains unclear. For future studies, it should be recommended that a quantitative method is implemented, as established in other examinations [22, 23, 24, 25]. Moreover, there remains a potential risk of contamination of the tested tool, such as through hair cosmetics or by contaminated hands. A random sample of tools (12.1%) was subjected to re‐testing and yielded a reproducibility rate of 100%. Thus, this is not considered to be a problem in this study.

## 5 Conclusion

This paper is the first to present nickel and cobalt test results regarding hairdressing tools in German barbershops. The results align with previous findings within hairdressing salons; also, in the sense that nickel release is more prevalent than cobalt release in hairdressing tools. Accordingly, barbers are potentially exposed to nickel and—to some extent—cobalt in their daily working life. This can lead to elicitation of allergic contact dermatitis in already sensitised workers. Also, the risk of occupational sensitisation cannot be ruled out, even though it can be assumed that this is less likely [17]. The novel findings should find entrance into preventive (health education) measures, such as awareness training. Further, and most importantly, it is recommended that compliance with the EU nickel regulation is monitored more strictly by the respective authorities in order to better protect the users of hairdressing tools.

## Author Contributions

**Cara Bieck:** conceptualisation; data curation; formal analysis; investigation; methodology; project administration; supervision; visualisation; writing – original draft preparation. **Kirsten Koopmann:** data curation; formal analysis; investigation; writing – review and editing. **Antje Alberts:** formal analysis, writing – review and editing. **Valeska Buder:** formal analysis; writing – review and editing. **Grita Schedlbauer:** resources; writing – review and editing. **Albert Nienhaus:** resources; writing – review and editing. **Christoph Skudlik:** resources; writing – review and editing. **Swen Malte John:** conceptualisation; resources; supervision; writing – review and editing.

## Conflicts of Interest

The authors declare no conflicts of interest.

## Data Availability

The data that support the findings of this study are available from the corresponding author upon reasonable request.
